# The prevalence of underweight, overweight, obesity and associated risk factors among school-going adolescents in seven African countries

**DOI:** 10.1186/1471-2458-14-887

**Published:** 2014-08-28

**Authors:** Taru Manyanga, Hesham El-Sayed, David Teye Doku, Jason R Randall

**Affiliations:** Department of Community Health Sciences, University of Manitoba, Winnipeg, Canada; Department of Pediatrics, Faculty of Medicine, Suez Canal University, Suez, Egypt; Department of Population and Health, University of Cape Coast, Cape Coast, Ghana

**Keywords:** Underweight, Overweight, Obesity, Prevalence and risk factors

## Abstract

**Background:**

The burden caused by the coexistence of obesity and underweight in Low and Middle Income Countries is a challenge to public health. While prevalence of underweight among youth has been well documented in these countries, overweight, obesity and their associated risk factors are not well understood unlike in high income countries.

**Methods:**

Cross-sectional data from the Global School-based Student Health Survey (GSHS) conducted in seven African countries were used for this study. The survey used a clustered design to obtain a representative sample (n = 23496) from randomly selected schools. 53.6% of the sample was male, and participants ranged in age from 11-17 years old. Body Mass Index (BMI) was calculated using age and sex adjusted self-reported heights and weights. Classification of weight status was based on the 2007 World Health Organization growth charts (BMI-for-age and sex). Multivariable Logistic Regression reporting Odds Ratios was used to assess potential risk factors on BMI, adjusting for age, sex, and country. Statistical analyses were performed with Stata with an alpha of 0.05 and reporting 95% confidence intervals.

**Results:**

Unadjusted rates of being underweight varied from 12.6% (Egypt) to 31.9% (Djibouti), while being overweight ranged from 8.7% (Ghana) to 31.4% (Egypt). Obesity rates ranged from 0.6% (Benin) to 9.3% (Egypt). Females had a higher overweight prevalence for every age group in five of the countries, exceptions being Egypt and Malawi. Overall, being overweight was more prevalent among younger (≤12) adolescents and decreased with age. Males had a higher prevalence of being underweight than females for every country. There was a tendency for the prevalence of being underweight to increase starting in the early teens and decrease between ages 15 and 16. Most of the potential risk factors captured by the GSHS were not significantly associated with weight status.

**Conclusions:**

The prevalence of both overweight and underweight was relatively high, demonstrating the existence of the double burden of malnutrition among adolescents in developing countries. Several factors were not associated with weight status suggesting the need to explore other potential risk factors for overweight and underweight, including genetic factors and socioeconomic status.

## Background

Until recently, childhood and adolescent overweight and obesity were mostly associated with affluence [1, 2] and the developed world [3–5]. These conditions have however significantly increased in low and middle income countries (LMICs) over time [6–8]. As of 2011, childhood overweight remained highest in high income countries (15%), but increasing rapidly as shown by the estimated 7% and 5% prevalence in Africa and Asia respectively [9]. Overweight and obesity in children and adolescents now co-exist with underweight, stunting and wasting [10–13] which historically, were associated with the developing world. The paradox of these two extremes, often referred to as the “double burden of malnutrition” [13–15] co-existing and largely attributable to nutrition transition in LMICs [4, 16], is a challenge to public health [7].

The World Health Organization (WHO) considers childhood obesity a serious public health challenge of the 21st century due in part, to its association with numerous deleterious health outcomes [17, 18]. On the other hand, being underweight (also associated with negative health outcomes) [12, 19, 20] remains a significant problem in LMICs [21, 22] despite increased efforts to address it [23]. In 2009, the WHO estimated 155 million or one in 10 school-age (5 – 17 years old) children worldwide to be either overweight or obese. In Africa, the estimated prevalence of childhood overweight increased from 4% in 1990 to 7% in 2011, and is expected to reach 11% in 2025 [9], while underweight is projected to increase albeit at a slower pace from 24% in 1990 to 26.8% by 2015 [24]. Overall, as of 2004, the prevalence of overweight (including obesity) was 8.4% while for obesity alone the prevalence was 1.9% [25].

Overweight and Obesity are associated with sedentary behaviour [26], and over-nutrition [27, 28] while underweight is partly related to undernutrition [29]. Neither of them (underweight, overweight or obesity) is a desirable health status and constitutes the extremes of malnutrition. Specific factors captured in the GSHS such as food insecurity [1, 16], fast food and soft drink consumption [30], decreased level of parental involvement [31, 32] as well as less fruit and vegetable consumption [32–34] have previously been associated with unhealthy weight status in youth. Although there is no consensus definition [35], at its core, malnutrition [36] is primarily (but not exclusively) a function of the imbalance between caloric intake and expenditure but other factors can independently influence either the intake or expenditure of calories [13, 37, 38].

Because of the negative health outcomes associated with the double burden of malnutrition, it is especially important to fully understand its prevalence and associated factors in school-aged adolescents. Behind the first year of life, adolescence is the second most critical period of physical growth [39]. While prevalence and factors associated with adolescent overweight and obesity in the developed world have been well documented, the same is not true for the developing world. There have been few studies on weight status of school-aged African youth and none, to the best of our knowledge, have assessed underweight, overweight, and obesity comparing many LMICs using data derived from a standard survey. The purpose of this study was to evaluate the prevalence of underweight, overweight and obesity as well as associated risk factors among school going adolescents in seven African countries using cross sectional data from the Global School-based Student Health Survey (GSHS).

## Methods

### Study design and sample

This study was cross-sectional and used data from a survey collected using a clustered sample design. The survey was conducted in the African countries of Benin, Djibouti, Egypt, Ghana, Mauritania, Malawi, and Morocco, as part of the GSHS. This survey was developed and administered by the WHO and the Center for Disease Control (CDC). The survey collected data from school-attending youth in 43 developing countries. These surveys utilized a clustered sample design to obtain a representative sample of school-attending youth in the equivalent of junior and senior high school (usually aged 13-17) years old in these countries. Survey schools were selected randomly with a probability proportional to their enrolment size. Within each selected school, classrooms were selected to participate and all students in selected classes were offered the opportunity to voluntarily and anonymously participate. A total of 25815 students were surveyed in these seven African countries between 2006 and 2010. Response rate varied between 85% and 100% for schools and 73% and 98% for students across the seven African countries. Sample size varied across the countries with larger countries requiring larger samples to achieve a representative sample. More information on the procedures, methods and questionnaires involved in the GSHS can be found on the WHO website [40] (http://www.who.int/chp/gshs/en/).

### Measures and response coding

The primary outcomes for this study are: being obese, overweight, or underweight according to the guidelines set out by the WHO for children and adolescents [41, 42]. Self-reported height and weight were used to obtain Body Mass Index (BMI) and code individuals as being underweight, overweight or normal weight based on these WHO guidelines. These guidelines lay age and gender-specific cut-offs for being underweight, overweight, and obese. Several potential risk factors for being underweight or overweight were extracted from the data. These factors include food insecurity, fruit and vegetable consumption, fast food consumption, soft drink consumption, parental support, and several measures of physical activity. These variables and their coding are described in Table 1. Questions were asked in the language of instruction used in the classrooms surveyed; English, French, or Arabic. Information on the proportion of each country coded with each factor is located in Table 2.

**Table 1:** **Independent variable derivation from survey data**

Survey question/variable	Coding (value coded)*	Variable
***How old are you?***	<12– 16 years (coded categorically)	Age
***What is your sex?***	Male (1)	Sex
	Female (0)	
***How tall are you without your shoes on?***		Used to calculate BMI
***How much do your weight without your shoes on?***		Used to calculate BMI
***During the past 30 days, how often did you go hungry because there was not enough food in your home?***	Most of the time/always (1)	Food insecurity
	Never/rarely/sometimes (0)	
***During the past 30 days, how many times per day did you usually eat fruit? (examples of fruit provided)***	2+ times a day (1)	Fruit consumption
	Less than 2 times a day (0)	
***During the past 30 days, how many times per day did you usually eat vegetables? (examples of vegetables provided)***	3+ times a day (1)	Vegetable consumption
	Less than 3 times a day (0)	
***Students who were physically active for a total of at least 60 minutes per day on five or more days during the past seven days***	Yes (1)	Consistently active
	No (0)	
***Students who went to physical education (PE) class on three or more days each week during this school year***	Yes (1)	PE class
	No (0)	
***Students who did not walk or ride a bicycle to or from school during the past seven days***	Yes (1)	No walking/biking to school
	No (0)	
***During the past 30 days, how many times per day did you usually drink carbonated soft drinks, such as Coca-Cola, Sprite, Fanta, Fizzi, Moka, Youki, or tonic?***	1+ times a day (1) Less than 1 per day (0)	Soft drink
***During the past 7 days, on how many days did you eat food from a fast food restaurant, such as [country specific examples]?***	3+ days (1) Less than 3 days (0)	Fast food
***How much time do you spend during a typical or usual day sitting and watching television, playing computer games, talking with friends, or doing other sitting activities such as [country specific examples]?***	3+ hours per day (1) Less than 3 hours per day (0)	Sitting 3+ hours
**Parental Support Index:** created by adding the results of the coding below		Parental support
***During the past 30 days, how often did you parents or guardians check to see if your homework was done?***	Most of the time/always (1)	Parental homework checking
	Never/rarely/sometimes (0)	
***During the past 30 days, how often did your parents or guardians understand your problems and worries?***	Most of the time/always (1)	Parental understanding
	Never/rarely/sometimes (0)	
***During the past 30 days, how often did you parents or guardians really know what you were doing with your free time?***	Most of the time/always (1)	Parental knowledge of activity
	Never/rarely/sometimes (0)	

*Note: STATA uses a code of 0 as the baseline group for binary and categorical variables.

**Table 2:** **Sample characteristics by country**

Variable		Benin	Djibouti	Egypt	Ghana	Malawi	Mauritania	Morocco
	Code	Proportion						
Food insecurity	0	0.823	0.806	0.948	0.781	0.815	0.896	0.893
	1	0.177	0.194	0.052	0.219	0.185	0.104	0.107
Fruit consumption	0	0.668	0.343	0.301	0.266	0.476	0.682	0.472
	1	0.332	0.657	0.699	0.734	0.524	0.319	0.528
Vegetable consumption	0	0.827	0.392	0.151	0.254	0.763	0.724	0.593
	1	0.173	0.608	0.849	0.746	0.237	0.277	0.407
Consistently active	0	0.678	*	*	0.842	*	0.829	0.818
	1	0.322	*	*	0.158	*	0.171	0.182
PE class	0	0.743	*	*	*	*	0.700	0.676
	1	0.257	*	*	*	*	0.300	0.324
No walking/biking to school	0	0.894	*	0.504	0.647	*	0.683	0.696
	1	0.106	*	0.496	0.353	*	0.317	0.304
Soft drink	0	0.720	*	*	*	*	0.495	0.536
	1	0.280	*	*	*	*	0.505	0.464
Fast food	0	0.824	*	0.882	*	*	0.714	0.850
	1	0.176	*	0.118	*	*	0.286	0.150
Sitting 3+ hours	0	0.810	*	*	0.730	*	0.629	0.740
	1	0.190	*	*	0.270	*	0.371	0.260
Parental support index	0	0.291	0.350	0.350	0.310	*	0.368	0.378
	1	0.260	0.298	0.264	0.268	*	0.263	0.276
	2	0.222	0.213	0.209	0.234	*	0.190	0.200
	3	0.227	0.140	0.177	0.188	*	0.179	0.146

*Data not available for this variable from this country.

### Statistical analysis

Age standardization was needed to derive prevalence that could be compared between countries. Weighting was applied to adjust for age so that the estimates would represent the prevalence that would occur if each country had the same age distribution as the seven countries combined. After weighting was applied the prevalence and 95% CI for being under/overweight, and obese was estimated for both genders for each of the seven countries.

The prevalence of underweight, overweight, and obesity was calculated for each age and sex group for each of the seven countries. These results were used to produce two line graphs depicting the prevalence of overweight/underweight/obesity by age for each of the countries. For each age, the average of the two sexes was used for the combined prevalence in order to remove the bias introduced by different gender proportions among countries.

Logistic regression reporting odds ratios (ORs) was used to determine the relationship between potential risk factors and being either underweight, overweight or obese. That is, those that were underweight were compared to the rest of the population for the underweight analyses. For the overweight analyses, the overweight individuals were compared to the rest of the population (those underweight or normal weight). A sensitivity analysis was done to ensure that comparing each outcome of interest to the rest of the population would not produce results different from comparing each outcome to those categorized as being normal weight. 95% confidence intervals (95% CI) were obtained for the ORs. The significance of the variables was tested using the Wald test with an alpha of 0.05. Food insecurity, fruit consumption and vegetable consumption were entered in a logistic model along with age, sex, and country. Other risk factors were also tested individually, in a model that included age, sex, and country. These factors were fast food consumption, soft drink consumption, parental support, and several measures of physical activity (measures described in Table 1). There is the possibility of heterogeneous effects of these factors between the countries in these data. To test this, the regression analyses were done using a dummy variable for each country and risk factor combination. A Wald test was used in post-estimation after the regression to determine if estimates varied significantly between these dummy variables. A significant result would indicate that the effect of the variable was heterogeneous among the countries. All analysis was done using STATA® version 13 [43]. Since the data were obtained from non-random samples, all of the analyses were done using the surveyset option in STATA to account for the survey design.

### Ethics statement

Consistent with the GSHS study protocol [40] (http://www.who.int/chp/gshs/en/), questionnaires were administered to all eligible participants in an anonymous, voluntary manner. Written permission had been obtained from each participating school and from all classroom teachers. Parents were informed of the study prior to the survey date. This article utilized publically available data and is exempt from institution ethical review.

The surveys were conducted in coordination with the governments of the countries involved prior to the initiation of the survey [40, 44]. The WHO specifies that 2 years after the surveys, data sets and code books associated with the core GSHS questionnaire modules will be made available to the public on the GSHS website. GSHS protocol states that once the data is made publically available then anyone interested in developing publications using the data is free to do so (http://www.who.int/chp/gshs/policy/en/) [44]. As is required for use of these public data , individual country coordinators were contacted and informed about this study, prior to its submission for publication and have been acknowledged for their contributions to this study [44].

## Results

### Sample

The characteristics of the samples seven countries are described in Table 3. The size of the samples varied from 1711, for Djibouti, to 6155 for Ghana. A total of 23496 observations were available with information about age, sex and weight status. Missing weight and height data is fairly common in these samples and the percentages of missing data due to absent BMI data are also presented. Four of the countries are missing less than 10% of the sample. Egypt and Malawi are missing BMI information for 13.3% of their sample and Mauritania is missing 34.6% of the sample. For most countries there is near parity of sexes with the exception of Benin and Djibouti, where males comprise a notable majority of respondents. Average age varied from 13.2 in Egypt to 15.2 in Benin. Unadjusted rates of being underweight varied from 12.6% (Egypt) to 31.9% (Djibouti). Rates of being overweight varied from 8.7% (Ghana) to 31.4% (Egypt). Rates of obesity ranged from 0.6% (Benin) to 9.3% (Egypt).

**Table 3:** **Sample characteristics by country**

	Benin	Djibouti	Egypt	Ghana	Malawi	Mauritania	Morocco
Survey year	2009	2007	2006	2007	2009	2010	2010
N (% missing BMI)	2681 (2.2%)	1711 (9.7%)	5179 (13.3%)	6155 (5.8%)	2305 (13.3%)	2028 (34.6%)	5756 (6.9%)
Male	65.1%	57.1%	52.8%	52.4%	46.6%	47.4%	52.4%
Age: 12 or under	4.3%	4.1%	22.3%	10.7%	5.8%	6.1%	14.4%
13	7.0%	6.6%	45.3%	16.3%	26.6%	9.5%	22.2%
14	14.8%	18.2%	22.8%	21.8%	38.6%	20.0%	26.8%
15	24.6%	27.8%	7.3%	26.9%	28.4%	28.9%	20.8%
16+	49.3%	43.2%	2.3%	24.3%	0.7%	35.6%	15.9%
Underweight	17.5%	31.9%	12.6%	25.7%	21.1%	22.3%	24.0%
Normal weight	71.3%	49.4%	56.0%	65.6%	68.9%	53.4%	59.4%
Overweight*	11.2%	18.8%	31.4%	8.7%	10.0%	24.3%	16.6%
Obese	0.6%	5.2%	9.3%	1.0%	0.8%	3.4%	3.6%

*Including the obese.

### Adjusted rates of underweight, overweight and obesity

Due to variations in age and sex among the countries, age-adjusted prevalence of being underweight, overweight, and obese stratified by sex is presented for each country in Table 4. Even after adjusting for age and sex differences there remained significant variation among countries. The lowest rate for being underweight was found in Egyptian females (9.9%) and the highest in Ghanaian males (33.8%). Females are less likely to be underweight for every country at every age.

**Table 4:** **Adjusted prevalence of underweight, overweight, and obesity**

		Prevalence		
Country	Sex	Underweight% (95% CI)*	Overweight**% (95% CI)*	Obesity% (95% CI)*
Benin	Female	14.0 (8-20)	21.6 (16-27)	2.9 (0-1)
	Male	19.9 (15-24)	16.1 (13-19)	0.3 (0-1)
Djibouti	Female	20.1 (14-26)	29.6 (24-35)	9.4 (6-13)
	Male	30.8 (24-38)	20.1 (16-24)	5.5 (3-8)
Egypt	Female	9.9 (8-12)	28.2 (23-34)	7.6 (5-10)
	Male	15.7 (13-19)	28.2 (23-33)	8.6 (6-11)
Ghana	Female	18.3 (16-20)	13.3 (12-15)	1.5 (1-2)
	Male	33.8 (31-36)	6.7 (5-8)	0.8 (0-1)
Malawi	Female	12.4 (8-17)	14.4 (10-19)	1.1 (0-3)
	Male	24.4 (18-31)	15.9 (9-22)	1.6 (0-4)
Mauritania	Female	17.9 (11-24)	35.9 (27-45)	5.1 (2-9)
	Male	23.5 (17-30)	22.0 (16-28)	2.7 (1-4)
Morocco	Female	17.1 (12-22)	19.3 (17-22)	3.9 (3-5)
	Male	29.3 (23-35)	15.6 (11-20)	3.6 (2-5)

*Adjusted for age distribution.

**Including those meeting the criteria for obesity.

There is wide variation for the prevalence of being overweight, ranging from 6.7% in Ghanaian males to 35.9% in Mauritanian females. Females have a higher prevalence of being overweight for every age group in five of the countries, exceptions being Egypt and Malawi. In Egypt the rates are equal at 28.2% whereas in Malawi the rate is slightly lower for females, though not statistically significant. The highest rate of obesity is in Djiboutian females at 9.4%. The lowest occurrence is in Beninese males at 0.3%.

**Figure 1:**
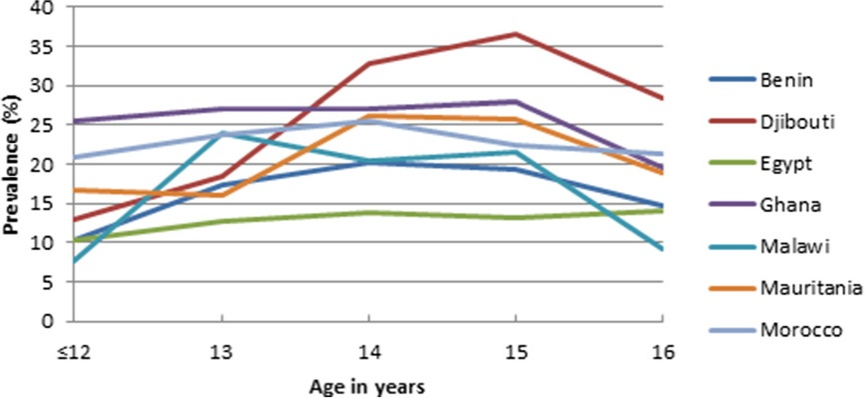
**Prevalence of being underweight.**

**Figure 2:**
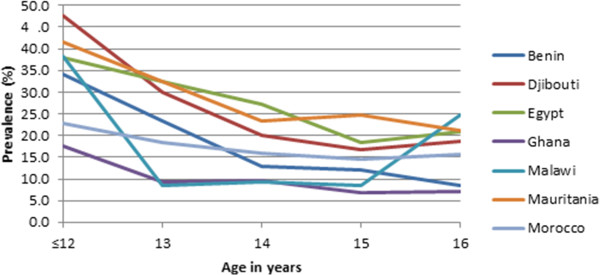
**Prevalence of being overweight.**

**Figure 3:**
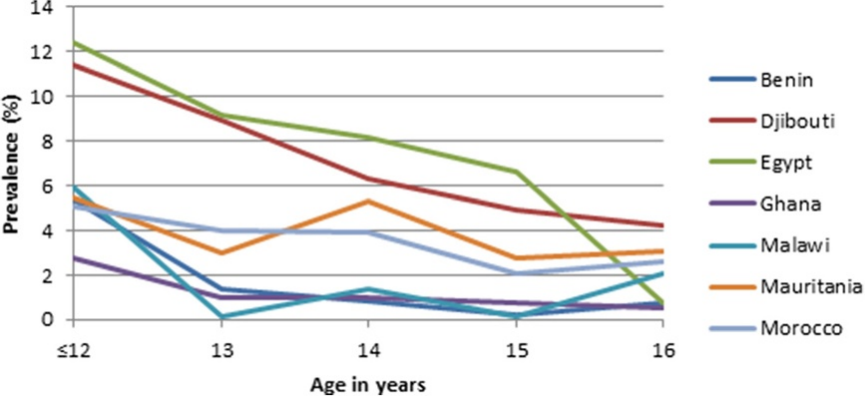
**Prevalence of obesity.**

Figure 1 shows the prevalence of being underweight for each country by age. There is a tendency for the prevalence of being underweight to rise, starting in the early teens and then decrease between ages 15 and 16. Figure 2 shows the prevalence of being overweight. Prevalence of being overweight decreases quickly at less than 13 years of age to age 14 and then largely remains stable with the notable exception of Malawi, where the rate increases significantly at age 16. Figure 3 shows the prevalence of obesity. There appears to be a downward trend in the prevalence of obesity with increasing age.

#### Risk factors for unhealthy weight

Results of the multivariable logistic regression are presented in Table 5. None of the potential risk factors measured in the questionnaires were found to be significantly related to being underweight. The only significantly related risk factor for being overweight was regular consumption of fruit (OR: 1.15; 95% CI: 1.01-1.31). There is significant variation in the occurrence of being overweight and obese across age groups. There is also a significant relationship between having 3+ days of physical education and being obese (OR: 1.51; 95% CI: 1.02-2.24).

**Table 5:** **Multivariable regression for weight risk factors**

		Underweight	Overweight	Obese
Multivariable model*		OR (95% CI)	OR (95% CI)	OR (95% CI)
Male sex		**2.03 (1.75-2.36)**	**0.76 (0.63-0.93)**	1.00 (0.76-1.34)
Age (ref = ≤12)	13	1.14 (0.90-1.45)	**0.76 (0.64-0.90)**	**0.69 (0.52-0.92)**
	14	1.21 (0.97-1.51)	**0.61 (0.48-0.77)**	**0.66 (0.48-0.90)**
	15	1.14 (0.90-1.45)	**0.48 (0.37-0.61)**	**0.43 (0.24-0.75)**
	16+	0.89 (0.68-1.17)	**0.50 (0.37-0.68)**	**0.41 (0.24-0.69)**
Food insecurity		0.93 (0.78-1.10)	1.06 (0.86-1.31)	1.02 (0.71-1.46)
Vegetables		0.97 (0.85-1.11)	0.97 (0.85-1.11)	0.95 (0.68-1.31)
Fruit		0.96 (0.83-1.09)	**1.15 (1.01-1.31)**	1.20 (0.94-1.53)
Risk factors with only partial data**				
Soft drinks		0.95 (0.80-1.13)	0.99 (0.77-1.27)	1.30 (0.88-1.92)
Fast food		1.09 (0.87-1.35)	1.12 (0.90-1.40)	1.28 (0.85-1.95)
Consistently active		0.93 (0.80-1.08)	1.23 (0.97-1.56)	1.13 (0.66-1.96)
Phys. Ed. 3+ days a week		0.97 (0.80-1.18)	1.20 (0.96-1.49)	**1.51 (1.02-2.24)**
Sitting 3+ hours a day		1.02 (0.88-1.19)	1.23 (0.97-1.56)	1.50 (0.94-2.40)
Does not walk/bike to school		0.94 (0.82-1.07)	0.98 (0.86-1.11)	1.00 (0.77-1.28)
Parental involvement index				
	1	0.95 (0.79-1.15)	1.17 (0.98-1.41)	1.05 (0.75-1.45)
	2	1.03 (0.87-1.23)	1.20 (0.99-1.45)	0.95 (0.72-1.26)
	3	0.97 (0.79-1.19)	**1.25 (1.01-1.56)**	1.14 (0.85-1.53)

*Sex, age, country, food insecurity, vegetables, and fruit adjusted for simultaneously.

**Adjusted for country, age and sex. Data not available for all countries for every variable.

Bolding indicates significance at p<0.05.

#### Assessment of heterogeneity of the risk factors

For most variables and outcomes there was no significant evidence for heterogeneity of effect among the countries. For the underweight outcome there were no significant tests for heterogeneity of effect for any of the variables measured. For the overweight outcome there was evidence for heterogeneity in effect for vegetable consumption (p = 0.0074), and not walking/biking to school (p = 0.023). Not walking/biking to school appears to reduce the odds of being overweight in Morocco (OR = 0.77; 95% CI: 0.61-0.97), while it appears to increase the odds for students in Ghana (OR = 1.28; 95% CI: 1.01-1.62). Vegetable consumption appears to increase the odds of being overweight in Mauritania (OR = 1.52; 95% CI: 1.09-2.11) and Malawi (OR = 1.57; 95% CI: 1.12-2.19) but has no effect in the other countries. For the obese outcome there was evidence for heterogeneity of effect for vegetable consumption (p = 0.026). Vegetable consumption significantly increases the odds of being obese in Mauritania (OR = 1.96; 95% CI: 1.01-3.83) and Malawi (OR = 3.03; 95% CI: 1.04-8.80) but no effect for the other countries.

#### Sensitivity analysis

Comparing different methods of grouping individuals for regression analysis did not find any significant differences between the results. Being male was no longer significantly associated with being overweight (OR = 0.76: 0.63-0.93), likely being male was associated with being underweight very significantly. Three other results went from being marginally not-significant to being significant. For being overweight, Sitting 3+ hours a day (OR = 1.25; 95% CI: 1.00-1.58) and Parental index involvement index category 2 were now significant. For being obese, sitting 3+ hours a day was now significant (OR = 1.58; 95% CI: 1.01-2.48). However the actual point estimates and confidence intervals did not change noticeably.

## Discussion

In the present study, the prevalence of being underweight in Benin, Djibouti, Egypt, Ghana, Malawi, Mauritania, and Morocco ranged from 12.6% in Egypt to as high as 31.9% in Djibouti. Being overweight was least prevalent in Ghana (8.7%) and highest in Egypt with 31.4%. Benin’s obesity rates were the lowest at 0.6% while Egypt had the highest at 9.3%. Egypt’s overweight prevalence was almost four times that of Ghana while having the lowest underweight rates; three times lower than Djibouti’s 31.9%. Overall, being overweight was more prevalent among younger (≤12) adolescents and decreased with age similar to findings of a previous study in Brazil [45]. The higher overweight and obesity prevalence in Egyptian youth may be indicative of nutrition transition occurring in the context of rapid urbanization [46] as well as an overall higher socio-economic status, which have been previously associated with overweight and obesity [47, 48].

Despite significant variation in the prevalence of unhealthy weights, results of this study are comparable to previous studies [14, 16, 49] showing the existence of the double burden of malnutrition in the seven African countries. Generally, the prevalence of overweight and obesity follow similar downward trends in all countries with the exception of Malawi where the trend seems to increase significantly after age 15. Inversely, underweight had an upward trend from early teens, peaking around age 14 with the exception of Djibouti whose underweight prevalence seem to peak at 15 years of age. The trend of underweight may be suggestive of family resource influence on weight status, with younger children dependent on their parents/guardians and from low socio-economic status [50] being more vulnerable and less so as they age and begin to fend for themselves [51]. Alternatively, this might be a result of early dropout from the schooling system by students with a higher propensity to be underweight, such as students from poorer socio-economic backgrounds [7].

Malawi’s overweight and obesity trends may be suggestive of different factors at play in Southern Africa than the more Northern countries. Overweight and obesity was most prevalent in Egypt followed by Mauritania, while lowest in Ghana. Although this does not clearly establish a North to South gradient, these rates are suggestive of regional differences in weight status among school-aged youth. A previous study, noted higher rates of overweight and obesity in North African children and adolescents and suggested this to be related to culture, diet and nutrition transition [25]. Regional differences in factors contributing to weight status is also evidenced by the presence of heterogeneity in some risk factors possibly related to varying urban vs. rural population distribution [21, 52] and levels of physical activity in these areas. The countries in North Africa are more developed and thus more likely to more sedentary employment as well as a move towards private car ownership culture, which reduces physical activity. Such countries may also more susceptible to Western influence in the area of influx of low cost of highly fatty food including refined cooking oil. Furthermore adolescent living in this countries are more likely to be exposed to the proliferation of technology based entertainments such as playing of computer game and watching of television at the expense of physical activity based ones. The differences may also be attributed to differences in lifestyle, culture and a shift from traditional foods [53].

Overall, our findings demonstrate a high prevalence of overweight status among African adolescents, surpassing the 11% overweight projection for the year 2025 [9]. The prevalence of underweight is comparable to findings of a previous study [24]. Given that in most African countries there are misconceptions about weight, for example having a “pot belly” is misconstrued as a sign of happiness, health and being wealthy [54–56], suggest that intervention is needed to counteract some of these misconceptions in order to prevent overweight and obesity among adolescents. Similar to previous studies [57–59], females had a higher overweight and obese prevalence than males while the reverse was true for underweight. This is different however to findings of previous studies in high income countries [60, 61] which showed a higher prevalence of overweight among adolescent boys compared to girls. In one of these studies, “obesity prevalence varied from 1.1% (Dutch and Danish girls) to 10.7% (Portuguese boys) and from 0.3% (Dutch girls) to 6.2% (Portuguese boys) respectively” [61]. Reasons for the different patterns in LMICs and High Income countries could include cultural beliefs about body image [62], and higher levels of physical activity including harder chores and manual labour among adolescent boys in LMICs than girls [62, 63]. Given such prevalence of unhealthy weights but no significant associations with potential risk factors, our findings seem to suggest that other factors not captured by the questions in the survey may be related to the weight status of these adolescents. This demonstrates the need for including survey questions that are sensitive to environmental, cultural, and contextual realities that exists in most of these African countries. This is necessary to enable accurate measurements of risk factors for unhealthy weights in African adolescents thus facilitate proper corrective and management strategies.

Of all the potential risk factors, only regular consumption of fruits, attending physical education more than 3 times a week and not walking/biking to school were associated with overweight and obese status respectively. This finding is somewhat counter-intuitive and is significantly different from previous studies [34, 60] which have shown the opposite. However, one study [48] showed that frequent involvement in sports was not associated with a lower BMI in Egyptian youth. This may be explained by the potential association of families’ higher socio-economic status (known to be a risk factor for overweight) [64] and regular or frequent fruit consumption as well as reported “higher participation” in sports. It is also possible that in the school setting, obese adolescents were required or encouraged to attend more physical exercise sessions. Alternatively, this could be an aberrant result, emanating from confounders or other factors not accounted for by the survey. Although none of the potential risk factors measured in the survey were associated with underweight, the results of our study highlights the existence of both extremes of unhealthy weights in school aged adolescents in seven African countries.

### Strengths and limitations

A specific strength of this study is that we used data from the GSHS with standardized sampling and survey questionnaires to compare adolescents from seven African countries. By presenting data from seven countries, this study provides a detailed comparative description of the prevalence of underweight, overweight and obesity among adolescents in these countries. The results of this study highlight important limitations of the GSHS questionnaire which may need to be modified to include validated and reliable anthropometric measures for ethnic minority children and adolescents.

The inherent inaccuracy of self-reported data and country specific differences limits the amount of inferences that can be drawn from the results of this study. Self-reported data can be especially unreliable when assessing dietary and physical activity behaviours of adolescents as highlighted by poor test-retest reliability in the Fijian version of the GSHS [65]. Another weakness is that in some of the seven countries, portions of the sample were missing data with the potential of introducing response-bias in the results of this study. Due to unavailability of other more reliable anthropometric measures, BMI values were calculated from the self-reported heights and weights which is often criticised for being unreliable [66–70] and not validated for ethnic minorities [71, 72], although other previous studies [73, 74], recommended its use. Using BMI can be especially problematic in paediatric populations where three different internationally recognized measures (CDC, IOTF, and WHO) of weight status [33, 75], are used and have been reported to result in different prevalence estimates of unhealthy weight status [33, 49, 75, 76].

## Conclusion

This study demonstrates the existence of the double burden of malnutrition among adolescents in all the seven African countries. Although the prevalence of obesity was relatively low in most samples, the findings of this study have important public health implications because unhealthy weight status (underweight, overweight and obesity) is not isolated to one region or country in Africa. Despite the high prevalence of underweight and overweight, several potential risk factors evaluated in this study were not significantly associated with the weight status of the participants, suggesting that other factors not captured by the survey may need to be explored.

Accurate estimates of the prevalence, and understanding factors associated with unhealthy weights is essential because of the importance of adolescence and the potential negative impacts of unhealthy weights in adulthood. There may be need for better survey design, reliability and validity assessment of the GSHS in different countries as well as prospective longitudinal studies that will investigate potential risk factors of unhealthy weights in African adolescents. This will enable assessments of potential effects of: genetics, environment and early exposures, which are known to be associated with weight development in childhood. It could also be argued that using robust anthropometric measures that are validated for these populations will yield more accurate and informative results, which will be beneficial in formulating policies and strategies for dealing with this problem. Regional differences in prevalence and associated risk factors are important to consider when policy initiatives are being formulated and implemented. Our findings also underscore the need to explore other potential risk factors for overweight and underweight among adolescents in developing countries. Future GSHS could consider including socio-economic indicators to enable exploring its relation with overweight and underweight.

## Abbreviations

LMICs: Low and Middle Income Countries
WHO: World Health Organization
BMI: Body Mass Index
GSHS: Global School-based Health Survey
CDC: Centres for Disease Control
IOTF: International Obesity Task Force
ORs: Odds Ratios
CI: Confidence interval.
