# Correction to: The prevalence of underweight, overweight, obesity and associated risk factors among school-going adolescents in seven African countries

**DOI:** 10.1186/s12889-019-6432-y

**Published:** 2019-01-30

**Authors:** Taru Manyanga, Hesham El-Sayed, David Teye Doku, Jason R. Randall

**Affiliations:** 1 Department of Community Health Sciences, University of Manitoba, Winnipeg, Canada; 2 Department of Pediatrics, Faculty of Medicine, Suez Canal University, Suez, Egypt; 3 Department of Population and Health, University of Cape Coast, Cape Coast, Ghana

**Correction to: BMC Public Health**

**https://doi.org/10.1186/1471-2458-14-887**

It was highlighted that in the original article [1] weight groupings were not derived using the mid-age cut-offs (e.g., those 14 years of age used the cut-offs derived for 14 years of age and not the more accurate 14 years 6 month cut-offs). The underweight cut-off used aligns with the cut-offs used for underweight status among older adolescents and adults, and not the weight-for-age cut-offs typically used among children under 10. With this Correction article, the authors want to notify the readers about this discrepancy and that the estimates are because of that slightly elevated. This error does not affect the interpretation of the results of the paper.
